# Safety of serotonin (5-HT3) receptor antagonists in patients undergoing surgery and chemotherapy: protocol for a systematic review and network meta-analysis

**DOI:** 10.1186/2046-4053-2-46

**Published:** 2013-06-28

**Authors:** Andrea C Tricco, Charlene Soobiah, Jesmin Antony, Brenda Hemmelgarn, David Moher, Brian Hutton, Sharon E Straus

**Affiliations:** 1 Li Ka Shing Knowledge Institute, St. Michael’s Hospital, 209 Victoria Street, East Building, Room 716, Toronto, Ontario M5B 1T8, Canada; 2 Departments of Medicine and Community Health Sciences, University of Calgary, 2500 University Dr NW, Calgary, Alberta T2N 4Z6, Canada; 3 Clinical Epidemiology Program, Centre for Practice-Changing Research, Ottawa Hospital Research Institute, 725 Parkdale Ave, Ottawa, Ontario K1Y 4E9, Canada; 4 Department of Geriatric Medicine, University of Toronto, 27 Kings College Circle, Toronto, Ontario M5S 1A1, Canada

## Abstract

**Background:**

Serotonin (5-HT3) receptor antagonists are a class of antiemetic medications often used to prevent nausea and vomiting among patients undergoing chemotherapy, radiotherapy or surgery. However, recent studies suggest that these agents might be associated with increased cardiac harm. To examine this further, we are proposing to conduct a systematic review and network meta-analysis on the comparative safety of 5-HT3 receptor antagonists among patients undergoing chemotherapy or surgery.

**Methods/Design:**

Studies reporting one or more safety outcomes of interest for 5-HT3 receptor antagonists compared with each other, placebo, and/or other anti-emetic agents (for example, benzamides, phenothiazines, butyrophenones, antihistamines, and anticholinergics) among children and adult patients undergoing surgery or chemotherapy will be included. Our primary outcome of interest is arrhythmia. Our secondary outcomes include cardiac death, QT prolongation, PR prolongation, all-cause mortality, nausea, and vomiting. We will include experimental studies, quasi-experimental studies (namely controlled before-after and interrupted time series), and observational studies (namely cohort studies). We will not limit inclusion by publication status, time period, duration of follow-up or language of dissemination.

Electronic databases (for example, MEDLINE, EMBASE) will be searched from inception onwards. These main searches will be supplemented by searching for difficult to locate and unpublished studies, such as dissertations, and governmental reports. The eligibility criteria will be pilot-tested and subsequently used to screen the literature search results by two reviewers in duplicate. A similar process will be followed for full-text screening, data abstraction, and risk of bias/methodological quality appraisal. The Cochrane Risk of Bias tool will be used to appraise experimental and quasi-experimental studies, and cohort studies will be assessed using the Newcastle Ottawa Scale. If the data allows, random effects meta-analysis and a network (that is, mixed treatment comparisons) meta-analysis will be conducted. All analyses will be conducted separately for different study designs, patient populations (for example, children and adults), and reason for administering 5-HT3 receptor antagonists (for example, post-surgery and chemotherapy).

**Discussion:**

Our results will help inform patients, clinicians, and health policy-makers about the potential safety concerns, as well as the comparative safety, of using these antiemetic agents.

**Trial registration:**

**PROSPERO registry number:**CRD42013003564

## Background

One of the most distressing symptoms for patients undergoing both surgery and chemotherapy is nausea and vomiting [1,2]. Nausea and vomiting has a significant impact on quality of life [3,4], and can lead to malnutrition [4], inability to respond to treatment [4], and an increased length of hospitalization [5]. Among patients undergoing surgery, postoperative nausea and vomiting has also been associated with pulmonary complications and wound dehiscence [6]. Most patients undergoing chemotherapy experience nausea and vomiting [7], while the occurrence of postoperative nausea and vomiting among patients following some surgical procedures can be as high as 70% [8].

Serotonin (5-HT3) receptor antagonists were introduced as antiemetic medications, as they inhibit vagal nerves in the central nervous system and intestinal mucosa that trigger the emetic reflex [9]. First-generation 5-HT3 receptor antagonists include ondansetron (brand name Zofran), dolasetron (brand names Anzemet, Anemet), and granisetron (brand names Sancuso, Kytril, Kevatril). Palonosetron (brand names Aloxi, Alexi) is a second-generation receptor antagonist [10]. These agents may be administered orally, subcutaneously, or intravenously.

Previous systematic reviews have found that 5-HT3 receptor antagonists are effective for postoperative and chemotherapy-induced nausea and vomiting [7,11-13]. As such, 5-HT3 receptor antagonists are recommended as the first-line treatment in chemotherapy-induced nausea and vomiting in adults and children [14]. They are also recommended for those patients undergoing surgery who are most at risk of postoperative nausea and vomiting [15,16].

Although 5-HT3 antagonist receptors might effectively reduce nausea and vomiting, concerns have been raised that these agents might be associated with cardiac risk. Some evidence suggest that 5-HT3 antagonist receptors prolong the QT interval (measured using electrocardiogram (ECG)) [17,18], which is associated with an increased risk of serious ventricular arrhythmias (for example, torsades de pointes). *In vitro* studies indicate that 5-HT3 antagonists block voltage-dependent sodium channels and hERG-potassium channel (cardiac ion channels), yet the magnitude and type of ECG change may differ for a particular drug, suggesting that the comparative safety of these medications is an important factor to examine. This is especially the case when factors that may impact blood levels of these agents are also present [19]. This information cannot be gleaned from previous systematic reviews of 5-HT3 receptor antagonists [7,11-13], as cardiac safety was not fully examined. As such, we are conducting this systematic review to determine the comparative safety of 5-HT3 receptor antagonists among patients undergoing chemotherapy or surgery. This research question was posed by policy makers in Canada to inform their decision-making related to these agents.

## Methods/Design

This is a protocol for a systematic review and network meta-analysis, which was registered with the PROSPERO database (CRD42013003564). Our reporting conforms to guidance put forth by the preferred reporting items for systematic reviews and meta-analyses protocols (or PRISMA-P) initiative [20].

### Eligibility criteria

We will include studies of adults (aged ≥18 years) and children undergoing surgery or chemotherapy who are administered first-generation and second-generation 5-HT3 antagonist receptors. We will explore the safety of each of the 5-HT3 antagonists against each other, against placebo, and/or other antiemetic agents, including benzamides, phenothiazines, butyrophenones, antihistamines, anticholinergics, and neurokin1 (NK1) antagonists. We will include studies of monotherapy of any dose, formulation, and duration. We will exclude studies that only include patients undergoing radiation therapy.

We will limit the systematic review to experimental studies (randomized clinical trials (RCTs), quasi-RCTs, non-RCTs), quasi-experimental studies (interrupted time series, controlled before and after studies), and observational (cohort) studies. The primary outcome of interest is arrhythmia. Secondary outcomes include sudden cardiac death, QT prolongation, PR prolongation, all-cause mortality, nausea (for example, the number of patients experiencing nausea or retching), vomiting (for example, the number of patients experiencing vomiting), and delirium.

Study inclusion will not be limited by publication status, time period, duration of follow-up, or language of dissemination. Studies will be excluded if they are animal studies or if there are no quantitative data to abstract (for example, letters, or commentaries). We will use Google Translate to determine eligibility and ScienceDocs, Inc. for translating languages in which the team is not fluent. A draft eligibility form is presented in Additional file 1 for both level 1 screening (titles and abstracts) and level 2 screening (potentially relevant full-text articles).

### Information sources and literature search

We will search the following electronic databases from inception onwards using medical subject headings (MeSH) and text words related to 5-HT3 receptor antagonists for nausea and vomiting: MEDLINE, EMBASE, and the Cochrane Central Register of Controlled Trials. We will supplement this search by conducting searches for grey literature (that is, those that are difficult to locate, or unpublished material) using guidance from the Canadian Agency for Technologies in Health (CADTH) [21]. Specifically, we will search public health, drug regulatory and trial registry websites (such as, Public Health Agency of Canada, Health Canada, Food and Drug Administration (FDA), and Clinical Trials.gov), websites of organizations that produce guidelines (such as, CADTH, Centre for Disease Control and Prevention, World Health Organization (WHO), Agency for Health Research and Quality, and the National Institute for Health and Clinical Excellence), trial protocol registries (such as, the WHO International Clinical Trials Registry Platform), and conduct general Internet searches in Google. Literature saturation will be ensured by searching the authors’ personal files, contacting 5-HT3 manufacturers, scanning the reference lists of included studies and relevant reviews, forward citation searching using Google, Scopus and Web of Science, and contacting experts in the field (such as, clinicians, and researchers).

All literature searches will be conducted by an experienced librarian. The main search strategy (MEDLINE) will be peer-reviewed by another librarian using Peer Review of Electronic Search Strategies (PRESS) [22]. The draft literature search for the main search strategy can be found in Additional file 1.

### Study selection process

To ensure high inter-rater reliability, a training exercise will be conducted prior to commencing screening. Using the predefined inclusion and exclusion criteria outlined in Additional file 1, a random sample of 50 titles and abstracts (citations) from the literature search will be screened by the systematic review team. We will calculate inter-rater agreement for study inclusion using the kappa statistic [23]. If poor-to-moderate agreement is observed (that is, kappa statistic ≤0.6), the eligibility criteria will be revised, as necessary. Subsequently, each citation will be screened by two reviewers in duplicate. Conflicts will be resolved by team discussion. A similar process will be followed for screening potentially relevant full-text articles. All levels of screening will be conducted using our online SysRev Tool, proprietary software developed by members of our group at the Li Ka Shing Knowledge Institute of St Michael’s Hospital [24].

### Data items and data collection process

We will abstract data on study characteristics (for example, study design, year of study conduct, sample size, setting, country of study conduct, intervention and comparator), participant characteristics (for example, number of patients, mean age and standard deviation, type and duration of chemotherapy, type of cancer, type and duration of surgery, type of anesthesia, history of previous conduction abnormality, use of diuretics, steroids or anti-arhythmics, co-morbidities, and metabolic abnormalities), and outcome results (for example, arrhythmia, and QT prolongation) for the following time points: 6, 12, and 24 months, as well as the longest duration of follow-up. Specific details on the interventions will be abstracted, including type of 5-HT3 receptor antagonist, dose, duration of use, cumulative dose and formulation.

Prior to commencing data abstraction, we will pilot-test a draft data abstraction form using a random sample of 10 included studies. When at least moderate agreement is observed (that is, kappa statistic ≤0.6), the data will be abstracted in duplicate by two reviewers. We will ensure that data are not counted twice by sorting through major publications and their respective companion reports, which include data from the same study group.

### Methodological quality/risk of bias appraisal

We will appraise the risk of bias of RCTs, quasi-RCTs, non-RCTs, interrupted time series, and controlled before-after studies using the Cochrane Effective Practice and Organisation of Care Risk of Bias Tool [25]. We will appraise the methodological quality of cohort studies using the Newcastle-Ottawa Scale [26]. Specific to studies reporting harms, the McHarm quality rating tool will be used [27]. We will assess for publication bias using funnel plots [28].

### Synthesis of included studies

We will first describe our systematic review results by reporting study characteristics, patient characteristics, risk of bias/methodological quality results, and frequencies of outcomes across the included studies. It is anticipated that we will have sufficient data to calculate pooled estimates of effects, which will be derived using a random-effects model [29]. We will ensure that the 95% confidence intervals can be derived using a normal distribution. Separate meta-analyses will be conducted by age category (that is, children versus adults) and patient population (that is, surgery versus chemotherapy).

Prior to conducting the meta-analysis, we will assess for three different types of heterogeneity: 1) clinical, 2) methodological, and 3) statistical heterogeneity. Clinical heterogeneity will be judged based on the clinical insight of the team. Similarly, methodological heterogeneity will be assessed by examining the included study characteristics and using our methodological judgment. Statistical heterogeneity will first be assessed by visually inspecting the forest plots. Subsequently, we will quantitatively assess heterogeneity using the I^2^ statistic [30]. A meta-regression analysis will be considered if at least 10 studies are available [31] and extensive heterogeneity is identified (for example, I^2^ statistic ≥60%) [30]. The meta-regression analysis will explore the influence of important factors, including children aged <3 years versus ≥3 years (as the latter group might have a higher incidence of postoperative nausea); type of chemotherapy (for example, use of cardiotoxic chemotherapy); type of anesthesia (for example, use of volatile anesthetics such as sevoflurane and isoflurane); type of surgery (for example, abdominal versus thoracic); use of steroids (for example, dexamethasone), diuretics, or anti-arrhythmics; history of conduction abnormality; and presence of metabolic abnormality (for example, hypo/hyperkalemia, hypo/hypercalcemia) on the meta-analysis results. These analyses will be conducted using SAS Version 9.2 [32].

Missing data (for example, measures of variance, such as standard deviations and standard errors) will be imputed using established methods [33].The influence of our imputations will be examined through a sensitivity analysis [34]. This approach entails imputing missing data under a missing-at-random assumption, and then reweights the imputed data to allow for examining the missing data under a nonrandom selection.

We anticipate that the data will allow us to conduct a network (that is, mixed treatment comparisons) meta-analysis, and our above-described explorations of heterogeneity will be helpful in assessing the key assumptions of homogeneity and similarity. The network meta- analysis, to be conducted in WinBUGS [35], will allow us to rank the effectiveness and safety of the different 5-HT3 medications [36]. Median rankings will be used as point estimates of the safety for 5-HT3 receptor antagonists. The network meta-analysis will be conducted using a random effects model including both direct and indirect treatment comparisons [36], as well as data from experimental, quasi-experimental, and cohort studies. To reflect the uncertainty of summary estimates, 95% credible intervals (CIs) will be calculated using the 2.5 and 97.5 percentiles obtained via Monte Carlo simulation of 100,000 iterations. These will be interpreted in the same manner as the 95% confidence intervals usually obtained through meta-analysis [36]. Model convergence will be assessed by examining trace and history plots, as well as by calculating the Gelman Rubin statistic [37]. The consistency of our results will be conducted by comparing our frequentist meta-analysis results to those obtained via network meta-analysis. This will also be assessed statistically, using node-splitting and back-calculation statistical methods [38,39].

Finally, we will conduct multiple sensitivity analyses to ensure that our results are robust. We will examine the impact of studies with high risk of bias/methodological quality, studies with high attrition rates, average adherence between groups, timing of receipt of the 5-HT3 antagonist, usage and formulation of the antiemetic, inclusion of quasi-experimental and observational studies in the analyses, as well as different priors for variance parameters included in the Bayesian meta-analysis [36].

## Discussion

This is the first systematic review that we are aware of to specifically focus on the cardiac safety of 5-HT3 receptor antagonists for postoperative and chemotherapy-induced nausea and vomiting, which includes study designs above and beyond RCTs. Due to the safety concerns of these agents, regulatory actions have been taken. For example, dolasetron is contraindicated for any use in children and for postoperative nausea and vomiting in adults in Canada. If 5-HT3 receptor antagonists truly are associated with a prolonged QT interval, and if this association is a class effect (that is, the risk of an adverse event is similar across all 5-HT3 antagonists), it could lead to limitations in use of these agents in adults and children. Alternatively, it could lead to a need for regular ECG monitoring of the patients who use these agents, which would inflict cost to the system and burden to the patients. Our systematic review will provide answers to these pertinent issues at hand.

We have developed a multi-faceted, knowledge translation strategy, which will be tailored to the relevant end users and will be finalized once the results are available. Our strategy may include presenting our findings at relevant conferences (for example, American Association for Cancer Research), and publishing our results in open access journals. We will also compile executive summaries tailored to stakeholder groups, such as clinicians and policy-makers. We will disseminate our results through newsletters of interested organizations, such as the Drug Safety and Effectiveness Network in Canada, and the Canadian Cancer Society. We will also convene dissemination dialogues with key stakeholders, including policy-makers, patients, and clinicians. Lastly, we will target social/mass media, to enhance mainstream uptake.

## Abbreviations

5-HT3: Serotonin; CADTH: Canadian agency for technologies in health; CI: Confidence interval; ECG: Electrocardiogram; FDA: Food and Drug Administration; MeSH: Medical subject headings; NK1: Neurokin 1 receptor; PRESS: Peer review of electronic search strategies; PRISMA-P: Preferred reporting items for systematic reviews and meta-analyses protocols; RCT: Randomized clinical trial; WHO: World Health Organization.

## Competing interests

The authors report no conflicts of interests.

## Authors’ contributions

ACT conceived the study, designed the study, helped obtain funding for the study, and helped write the draft protocol. CS registered the protocol with the PROSPERO database and edited the draft protocol. JA edited the draft protocol. BH, DM, and BH helped conceive the study, and edited the protocol. SES conceived the study, designed the study, obtained the funding, and helped write the draft protocol. All authors read and approved the final protocol.

## Supplementary Material

Additional file 1 Appendix. Click here for file
