# Supplementary material for: Safety of serotonin (5-HT3) receptor antagonists in patients undergoing surgery and chemotherapy: protocol for a systematic review and network meta-analysis
Source: Syst Rev. 2013 Jun 28;2:46. doi: 10.1186/2046-4053-2-46 (PMC3702491; doi:10.1186/2046-4053-2-46)
Supplement: Additional file 1 — Appendix. [file 2046-4053-2-46-S1.docx]

**Appendix 1: Draft Eligibility Criteria**

Level 1 screening

1. Does this study include adult patients (aged ≥ 18 years) or children who are undergoing chemotherapy or surgery?

YES____ NO____ UNCLEAR____

1. Is this an experimental, quasi-experimental or cohort study?

YES____ NO____ UNCLEAR____

1. Are patients treated with the following 5-HT3 receptor inhibitors?

YES____ NO____ UNCLEAR____

| **Generic name** | **Trade name(s)** |
| --- | --- |
| Ondansetron | Zofran |
| Dolasetron | Azemet, Anemet |
| Granisetron | Sancuso, Kytril, Kevatril |
| Palonosetron | Aloxi, Alexi |

1. Does the study compare a 5-HT3 receptor inhibitor with another 5-HT3 receptor inhibitor, placebo, and/or other antiemetic agents?

YES____ NO____ UNCLEAR____

- If you answer NO to any of these questions, the citation/study will be excluded. All othercitations will be included.Level 2 screening

1. Does this study include adult patients (aged ≥ 18 years) or children who are undergoing chemotherapy or surgery?

YES____ NO____ UNCLEAR____

1. Is this an experimental, quasi-experimental or cohort study?

YES____ NO____ UNCLEAR____

1. Are patients treated with the following 5-HT3 receptor inhibitors?

YES____ NO____ UNCLEAR____

| **Generic name** | **Trade name(s)** |
| --- | --- |
| Ondansetron | Zofran |
| Dolasetron | Azemet, Anemet |
| Granisetron | Sancuso, Kytril, Kevatril |
| Palonosetron | Aloxi, Alexi |

1. Does the study compare a 5-HT3 receptor inhibitor with another 5-HT3 receptor inhibitor, placebo, and/or other antiemetic agents?

YES____ NO____ UNCLEAR____

1. Does the study report at least one of the following outcomes?

Arrhythmia, sudden cardiac death, QT prolongation, PR prolongation, all-cause mortality, nausea, or vomiting.

YES____ NO____ UNCLEAR____

- If you answer NO to any of these questions, the citation/study will be excluded. All other full-text articles will be included.

**Appendix 2: Draft literature search for MEDLINE**

1 Ondansetron/ [ Ondansetron ]

2 ondansetron.mp.

3 zofran.mp.

4 SN-307.mp.

5 SN307.mp.

6 GR38032F.mp.

7 GR-38032F.mp.

8 GR C50775.mp.

9 99614-02-5.rn. [ CAS Registry ]

10 bryterol.mp.

11 cedantron.mp.

12 ceramos.mp.

13 emeset.mp.

14 modifical.mp.

15 narfoz.mp.

16 onsia.mp.

17 sakisozin.mp.

18 vomceran.mp.

19 zofrene.mp.

20 zefron.mp.

21 zophron.mp.

22 zophran.mp.

23 zuplenz.mp.

24 zophren.mp.

25 zudan.mp.

26 Granisetron/ [ Granisetron ]

27 granisetron$.mp.

28 kytril.mp.

29 BRL-43694.mp.

30 BRL43694.mp.

31 109889-09-0.rn. [ CAS Registry ]

32 apf 530.mp.

33 eutrom.mp.

34 granicip.mp.

35 granisol.mp.

36 kevatril.mp.

37 sancuso.mp.

38 taraz.mp.

39 dolasetron.mp. [ Dolasetron ]

40 anzemet.mp.

41 anemet.mp.

42 zamanon.mp.

43 MDL 73,147EF.mp.

44 MDL-73147EF.mp.

45 dolasetron.rn. [ CAS Registry ]

46 palonosetron.mp.

47 135729-61-2.rn. [ CAS Registry ]

48 onicit.mp.

49 aloxi.mp.

50 2-Qhbiqo.mp.

51 Serotonin 5-HT3 Receptor Antagonists/

52 5ht3.mp.

53 5-HT3.mp.

54 "5-Hydroxytryptamine-3 receptor antagonist?".mp.

55 "serotonin type 3 receptor antagonist$".mp.

56 "5-hydroxytryptamine-3 antagonist$".mp.

57 or/1-56

58 chemotherap$.mp. [ chemotherapy ]

59 chemo-therap$.mp.

60 Antineoplastic Combined Chemotherapy Protocols/

61 exp Antineoplastic Agents/

62 canc$.mp. [ cancer / oncology ]

63 carcinoma$.mp.

64 tumo?r$.mp.

65 neoplasm$.mp.

66 on?olog$.mp.

67 surger$.mp. [ surgery ]

68 surgical$.mp.

69 su.fs.

70 exp Surgical Procedures, Operative/

71 Nausea/ [ nausea and vomiting ]

72 Vomiting/

73 nause$.mp.

74 vomit$.mp.

75 emesis.mp.

76 PONV.mp.

77 "Postoperative Nausea and Vomiting"/

78 or/58-77

79 57 and 78

80 exp Animals/ not (exp Animals/ and Humans/)

81 79 not 80
